# Newly diagnosed multiple myeloma patients with CD56 expression benefit more from autologous stem cell transplantation

**DOI:** 10.1186/s12885-022-10382-0

**Published:** 2022-12-23

**Authors:** Chuanying Geng, Huixing Zhou, Huijuan Wang, Yanchen Li, Yun Leng, Zhiyao Zhang, Yuan Jian, Guangzhong Yang, Wenming Chen

**Affiliations:** Department of Hematology, Beijing Chao-Yang Hospital, Capital Medical University, Beijing, 100020 China

**Keywords:** Multiple myeloma, CD56, Autologous stem cell transplantation, Survival

## Abstract

**Background:**

Several studies showed that lack of CD56 expression was a poor prognostic factor for patients with newly diagnosed multiple myeloma (NDMM). However, other studies were not able to confirm the prognostic value of CD56 in NDMM. This study aimed to evaluate the prognostic value of CD56 expression for patients with NDMM who received autologous stem cell transplantation (ASCT).

**Methods:**

We retrospectively analyzed 370 patients with NDMM under 66 years old and the propensity score matching technique was used to reduce the bias between two groups.

**Results:**

CD56 expression was observed in 250 (67.6%) patients, and only half of transplant-eligible patients received ASCT for financial and adverse effects concerns after induction therapy. 54.8% (137/250) CD56 positive patients received ASCT; and 47.5% (57/120) CD56 negative patients received ASCT. Univariate and multivariate analyses showed that ASCT was correlated with longer overall survival (OS) (*p* < 0.001) and progression-free survival (PFS) (*p* < 0.001) for CD56 positive patients. However, ASCT had no impact on OS and PFS in univariate and multivariate analysis (*p* > 0.05). In the propensity score matching analysis, 186 CD56 positive patients were identified, 93 patients had received ASCT and 93 patients had no ASCT. Among 120 CD56 negative patients, 80 patients, 40 in each group, were identified. Among 186 matched CD56 positive patients, patients with ASCT had longer OS (87.6 vs.56.1 months, *p* = 0.049) and PFS (36.7 vs.30.9 months, *p* = 0.040). However, ASCT had no impact on OS and PFS for matched CD56 negative patients (*p* > 0.05).

**Conclusions:**

These results demonstrated that ASCT may improve OS and PFS of CD56 positive patients and had no impact on survival of CD56 negative patients.

## Background

Multiple myeloma (MM) is a common hematological malignancy which originates from clonal plasma cells [1]. MM remains an incurable disease until nowadays, and autologous stem cell transplantation (ASCT) is the standard treatment for newly diagnosed MM (NDMM), despite the advent of novel agents [2, 3]. For a heterogenous disease, survival interval for patients varies significantly, from a few months to more than 10 years [4]. It is important to identify prognostic factors for MM. During several decades, it has developed many useful prognostic factors, including Durie-Salmon (DS) stage, International Staging System (ISS), lactate dehydrogenase (LDH) level, high-risk cytogenetics abnormalities [5]. Recently, Revised ISS (R-ISS) stage system combined ISS stage system with cytogenetic abnormalities plus lactate dehydrogenase (LDH) to better predict outcomes of patients with MM [6]. It has been reported that certain immunophenotypes of plasma cells may impact MM prognosis and clinical characteristics. CD56 was an isoform of the neural cell adhesion molecule which was able to mediate the adhesion of MM cells to the extracellular matrix. Studies showed that CD56 expression could be detected in 55 ~ 85% patients with MM [7–13]. CD56 expression was constant over the course of MM and it was significantly linked to the degree of both bone marrow and peripheral blood involvement [14]. CD56 expression by plasma cells also correlated with the presence of lytic bone lesions in MM [15]. Sahara N et al. [16] reported that MM with CD56 negative had a poor prognosis with higher chance of extramedullary disease, Bence Jones protein, renal insufficiency, thrombocytopenia, and plasma cell morphology. CD56 expression level was lower in advanced stages than earlier stages [13]. Lack of CD56 expression was a poor prognostic factor for patients with NDMM [7, 8, 17]. Expression of CD56 was associated with better response to bortezomib treatment and was a promising candidate biomarker for predicting response to therapeutic regimens contained bortezomib [18]. However, other studies reported that lack of CD56 expression was not risk factors for survival in patients with MM [10, 11, 19–21].

Almost all patients who received ASCT in our center achieved PR or better without serious complications and were under 66 years old. So, we retrospectively analyzed 370 patients with NDMM under 66 years old who obtained PR or better after induction therapy and had no serious complications in Beijing Chao-Yang Hospital, Capital Medical University. We found that ASCT may improve the overall survival (OS) and progression-free survival (PFS) of CD56 positive patients and had no impact on survival of CD56 negative patients in novel-agent era.

## Methods

### Patients

We recorded baseline data of NDMM patients in Beijing Chaoyang Hospital, Capital Medical University from February 1, 2011 to October 1, 2021 by searching the Electronic Medical Record System (EMRS). The International Myeloma Working Group (IMWG) criteria of MM was used to confirm patients with NDMM and all patients were followed up until March 1, 2022 [3]. We followed the patients through the EMRS without disturbing patients in any way. Bone marrow specimen testing was a routine examination for the diagnosis and evaluation of MM in our center. The CD56 expression was detected by flow cytometry. Whole bone marrow samples were stained using CD56 antibody for 15 mins at room temperature and stained cells were detected on a BD FACSCanto II (BD Biosciences). The gate was based on CD38/CD138 for plasma cells and the cutoff for expression of the CD56 was 20.0%. Cytogenetic abnormalities were detected by Fluorescence in situ hybridization (FISH), including t (4; 14), t (14; 16) and del17p13. All patients received induction therapy containing new drugs including bortezomib, lenalidomide, or thalidomide. After induction and ASCT, patients received consolidation therapy, followed by maintenance therapy with bortezomib, lenalidomide, or thalidomide. Patients who did not receive ASCT received consolidation and maintenance therapy after induction therapy. This study followed to the principles of the Declaration of Helsinki and was approved by the Medical Ethics Committee of Beijing Chaoyang Hospital.

### Response and outcome measures

Patient responses were confirmed according to the IMWG criteria [22]. The main indexes included stringent complete remission (sCR), complete remission (CR), very good partial remission (VGPR) and partial remission (PR) based on the assessment of serum and urine protein electrophoresis, immunofixation, serum-free light chain assay, and bone marrow aspiration and biopsy. If the patients had extramedullary disease at diagnosis, ^18^F-FDG PET/CT was necessary for response analysis. Primary endpoints were PFS and OS. The time from diagnosis to disease progression or death was defined as the estimated PFS, and the time from diagnosis to death from any cause or last exposure date was defined as the estimated OS. Patients who could not be followed up were censored at last contact.

### Statistical analysis

Statistical analysis was carried out through SPSS 23.0 software. Categorical variables were analyzed by Chi-square test or Fisher’s exact test. PFS and OS were estimated according to Kaplan-Meier method and the survival differences were compared by two-tailed log-rank test. The COX proportional hazards regression analyses were used to assess the prognostic impact, and results were reported as hazard ratios (HRs) with 95% confidence intervals (95% CIs). Propensity score matching techniques were used to balance the distribution of factors with prognostic value in previous studies or in this study. *P* values less than < 0.05 were considered statistically significant, and all tests were two-sided.

## Results

### Patient characteristics

A total of 370 patients with NDMM under 66 years old were enrolled, CD56 expression was detected in 250 (67.6%) patients and 194 (52.4%) patients received ASCT after induction therapy containing novel agents with 12 months. There were 137/250 (54.8%) and 57/120 (47.5%) received ASCT in CD56 positive and negative patients, respectively. Table 1 summarized the characteristics of 370 patients. The male-to-female ratio was 1.22 (203/167) and the median age was 56 (range 33–65) years old. The most common monoclonal protein was IgG type (50.0%) and 169 (45.7%) were at ISS stage III. All patients received induction therapy combining novel agents, 167 (45.1%) patients received bortezomib-based regimens, 30 (8.1%) combining immunomodulatory drugs (IMiDs), 173 (46.8%) combining bortezomib and IMiDs. After induction therapy, 194 (52.4%) patients received ASCT. As shown in Table 1, there were statistically significant differences between CD56 positive and negative patients in MM subtype, t (14; 16) and t (4; 14).

**Table 1:** Baseline clinical and biological characteristics of MM patients

Characteristics	all patients	CD56 positive	CD56 negative	*p* value
	*n* = 370	*n* = 250	*n* = 120	
	n (%)	n (%)	n (%)	
Sex				
Male	203 (54.9)	129 (51.6)	74 (61.7)	0.07
Female	167 (45.1)	121 (48.4)	46 (38.3)	
Median age (range) [years]	56 (33–65)	56 (33–65)	57 (33–65)	0.59
MM subtype				
IgG	185 (50.0)	137 (54.8)	48 (40.0)	0.00
IgA	73 (19.7)	54 (21.6)	19 (15.8)	
IgD	22 (5.9)	5 (2.0)	17 (14.2)	
Light chain only	78 (21.1)	48 (19.2)	30 (25.0)	
Non-secretory	12 (3.2)	6 (2.4)	6 (5.0)	
ISS stage				
I	78 (21.1)	46 (18.4)	32 (26.7)	0.11
II	123 (33.2)	90 (36.0)	33 (27.5)	
III	169 (45.7)	114 (45.6)	55 (45.8)	
Hemoglobin				
< 100 g/L	229 (61.9)	160 (64.0)	69 (57.5)	0.23
≥ 100 g/L	141 (38.1)	90 (36.0)	51 (42.5)	
Serum creatinine				
≤ 2 mg/dL	309 (83.5)	210 (84.0)	99 (82.5)	0.72
> 2 mg/dL	61 (16.5)	40 (16.0)	21 (17.5)	
Corrected serum calcium				
≤ 2.75 mmol/L	323 (87.3)	214 (85.6)	109 (90.8)	0.16
> 2.75 mmol/L	47 (12.7)	36 (14.4)	11 (9.2)	
Lactate dehydrogenase				
≤ 250 U/L	318 (85.9)	219 (87.6)	99 (82.5)	0.19
> 250 U/L	52 (14.1)	31 (12.4)	21 (17.5)	
Cytogenetic abnormalities by FISH				
del(17p13)				
abnormality	40 (10.8)	30 (12.0)	10 (8.3)	0.29
non-abnormality	330 (89.2)	220 (88.0)	110 (91.7)	
t(14; 16)				
abnormality	15 (4.1)	2 (0.8)	13 (10.8)	0.00
non-abnormality	355 (95.9)	248 (99.2)	107 (89.2)	
t(4; 14)				
abnormality	69 (18.6)	67 (26.8)	2 (1.7)	0.00
non-abnormality	301 (81.4)	183 (73.2)	118 (98.3)	
Induction regimes				
Bortezomib based	167 (45.1)	114 (45.6)	53 (44.2)	0.90
IMiD based	30 (8.1)	21 (8.4)	9 (7.5)	
Bortezomib and IMiD based	173 (46.8)	115 (46.0)	58 (48.3)	
ASCT				
Yes	194 (52.4)	137 (54.8)	57 (47.5)	0.19
No	176 (47.6)	113 (45.2)	63 (52.5)	

*Abbreviations*: *IMiD* Immunomodulatory, *ASCT* Autologous stem cell transplant

### Multivariate analysis for survival

Univariate analysis found six factors associated with OS and they were LDH > 250 U/L, CsCa > 2.75 mmol/L, del (17p13), t (14; 16), ISS III stage and ASCT. Multivariate analysis was performed for hemoglobin (HGB) < 100 g/L, serum creatinine (SCr) > 2 mg/dL, CD56, t (4; 14) and these six covariates. It was showed that ASCT was a favorable factor for OS (HR = 0.55, 95%CI: 0.37–0.82, *p* < 0.001) and PFS (HR = 0.62, 95%CI: 0.46–0.83, *p* < 0.001) of patients with NDMM in multivariate analyses (Table 2). Among CD56 positive patients, univariate analyses showed that ASCT was a favorable factor for OS (HR = 0.41, 95%CI: 0.26–0.67, *p* < 0.001) and PFS (HR = 0.57, 95%CI: 0.40–0.81, *p* < 0.001); the favorable effect of ASCT on OS (HR = 0.38, 0.23–0.64, *p* < 0.001) and PFS (HR = 0.60, 0.42–0.87, *p* = 0.001) was confirmed in multivariate analyses (Table 2). Among CD56 negative patients, univariate analyses showed that ASCT had no impact on PFS (*p* = 0.17) and OS (*p* = 0.53); and multivariate analyses also showed that ASCT had no effect on PFS (*p* = 0.21) and OS (*p* = 0.46) (Table 2).

**Table 2:** Cox analysis (univariate and multivariate) of ASCT

	all		CD56 positive		CD56 negative	
	*P* value HR (95% CI)		*P* value HR (95% CI)		*P* value HR (95% CI)	
Univariate						
OS	0.00	0.54 (0.37–0.79)	0.00	0.41 (0.26–0.67)	0.53	1.23 (0.65–2.35)
PFS	0.00	0.60 (0.45–0.80)	0.00	0.57 (0.40–0.81)	0.17	0.69 (0.41–1.17)
Multivariate						
OS	0.00	0.55 (0.37–0.82)	0.00	0.38 (0.23–0.64)	0.46	1.29 (0.66–2.53)
PFS	0.00	0.62 (0.46–0.83)	0.01	0.60 (0.42–0.87)	0.21	0.70 (0.40–1.22)

*Abbreviations*: *HR* Hazard ratio; *95% CI* 95%confidence interval, *ASCT* Autologous stem cell transplant

### Matched pairs of patients

Among CD56 positive patients, ASCT and non-ASCT patients were matched for age, ISS stage, HGB, SCr, CsCa, LDH, del (17p13), t (14; 16) and t (4; 14). A total of 186 patients were identified by propensity score matching technique, with 93 patients in each group. It was showed that there was no significantly difference in matched groups of ASCT and non-ASCT patients with respect to these characteristics (Table 3). Among CD56 negative patients, ASCT and non-ASCT patients were matched for above similar factors and 80 patients, 40 in each group, were identified. These two matched groups also had no difference in these factors (Table 3).

**Table 3:** Baseline clinical and biological characteristics of matched patients

Characteristics	CD56 positive		CD56 negative	
	ASCT	non-ASCT	ASCT	non-ASCT
	*n* = 93	*n* = 93	*n* = 40	*n* = 40
	n (%)	n (%)	n (%)	n (%)
Median age (range) [years]	56 (36–65)	57 (38–65)	57 (38–65)	57 (35–65)
ISS stage				
I	16 (17.2)	13 (14.0)	11 (27.5)	9 (22.5)
II	34 (36.6)	39 (41.9)	12 (30.0)	12 (30.0)
III	43 (46.2)	41 (44.1)	17 (42.5)	19 (47.5)
Hemoglobin				
< 100 g/L	57 (61.3)	62 (66.7)	20 (50.0)	24 (60.0)
≥ 100 g/L	36 (38.7)	31 (33.3)	20 (50.0)	16 (40.0)
Serum creatinine				
≤ 2 mg/dL	80 (86.0)	80 (86.0)	35 (87.5)	36 (90.0)
> 2 mg/dL	13 (14.0)	13 (14.0)	5 (12.5)	4 (10.0)
Corrected serum calcium				
≤ 2.75 mmol/L	82 (88.2)	80 (86.0)	37 (92.5)	36 (90.0)
> 2.75 mmol/L	11 (11.8)	13 (14.0)	3 (7.5)	4 (10.0)
Lactate dehydrogenase				
≤ 250 U/L	83 (89.2)	81 (87.1)	34 (85.0)	33 (82.5)
> 250 U/L	10 (10.8)	12 (12.9)	6 (15.0)	7 (17.5)
Cytogenetic abnormalities by FISH				
del(17p13)				
abnormality	10 (10.8)	12 (12.9)	2 (5.0)	1 (2.5)
non-abnormality	83 (89.2)	81 (87.1)	38 (95.0)	39 (97.5)
t(14; 16)				
abnormality	0 (0.0)	0 (0.0)	3 (7.5)	5 (12.5)
non-abnormality	93 (100.0)	93 (100.0)	37 (92.5)	35 (87.5)
t(4; 14)				
abnormality	26 (28.0)	25 (26.9)	0 (0.0)	0 (0.0)
non-abnormality	67 (72.0)	68 (73.1)	40 (100.0)	40 (100.0)

### Response analysis

All patients were monitored for best response after ASCT and consolidation therapy. Among the 186 matched CD56 positive patients, 73 patients (39.2%) achieved sCR, 24 (12.9%) CR, 55 (29.6%) VGPR, and 34 (18.3%) PR. Patients received ASCT had the higher sCR rate (48.4%) than those without ASCT (30.1%) in the matched groups (*p* < 0.001, Table 4). Among the 80 matched CD56 negative patients, 36 patients (45.0%) achieved sCR, 11 (13.8%) CR, 19 (23.8%) VGPR, and 14 (17.5%) PR. Patients received ASCT also had the higher sCR rate (67.5%) than those without ASCT (22.5%) in the matched groups (*p* < 0.001, Table 4).

**Table 4:** Best response rate of matched patients

Response	CD56 positive		CD56 negative	
	ASCT *	non-ASCT	ASCT *	non-ASCT
	*n* = 93	*n* = 93	*n* = 40	*n* = 40
	n (%)	n (%)	n (%)	n (%)
sCR	45 (48.4)	28 (30.1)	27 (67.5)	9 (22.5)
CR	15 (16.1)	9 (9.7)	6 (15.0)	5 (12.5)
VGPR	28 (30.1)	27 (29.0)	5 (12.5)	14 (35.0)
PR	5 (5.4)	29 (31.2)	2 (5.0)	12 (30.0)

*Abbreviations*: *sCR* Stringent complete response, *CR* Complete response, *VGPR* Very good partial response; *: *p* < 0.001

### Survival analysis

**Fig. 1:**
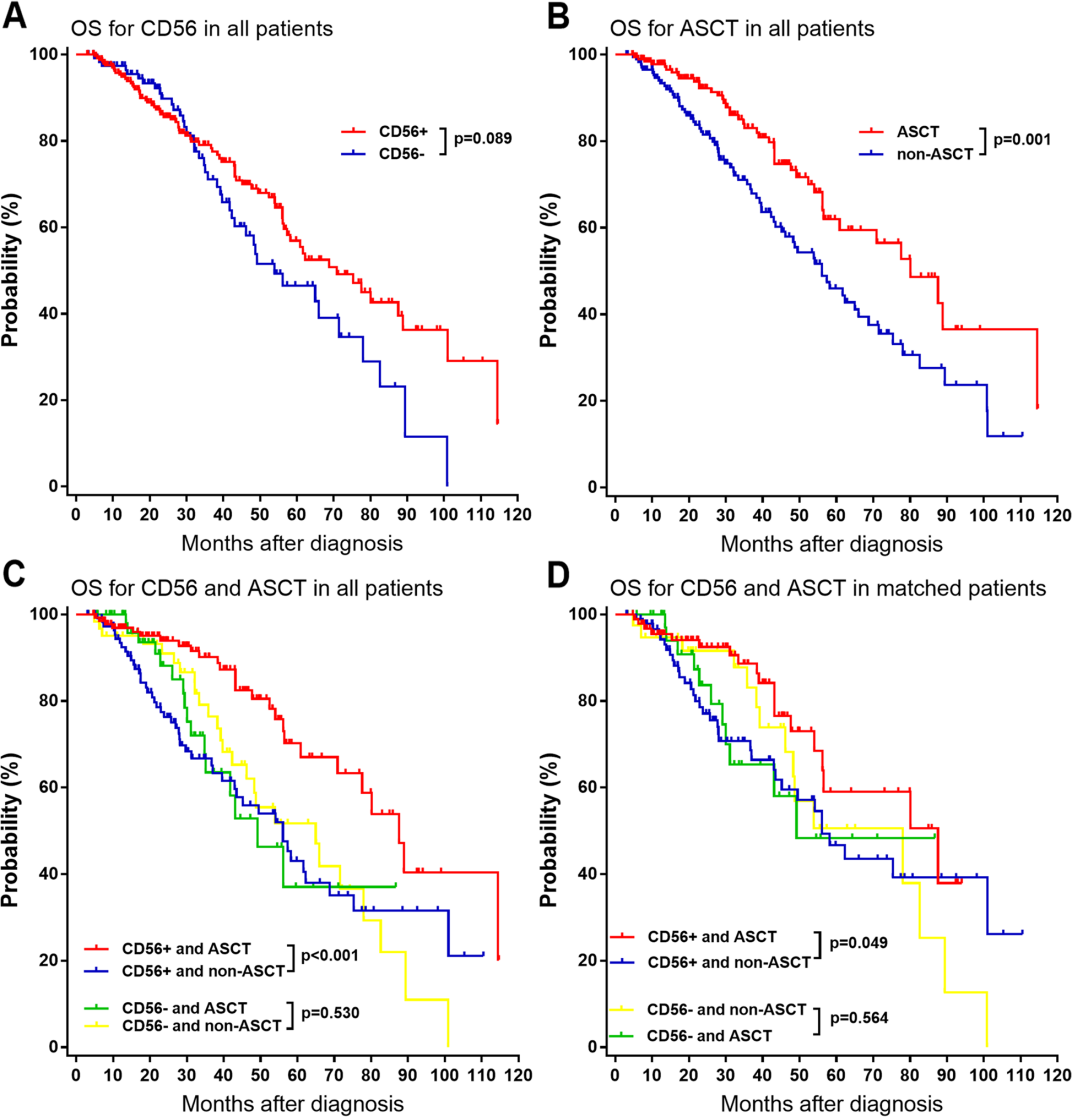
Kaplan-Meier survival curves on OS of patients with NDMM. **A** all patients. **B** all patients. **C** all patients. **D** matched patients

**Fig. 2:**
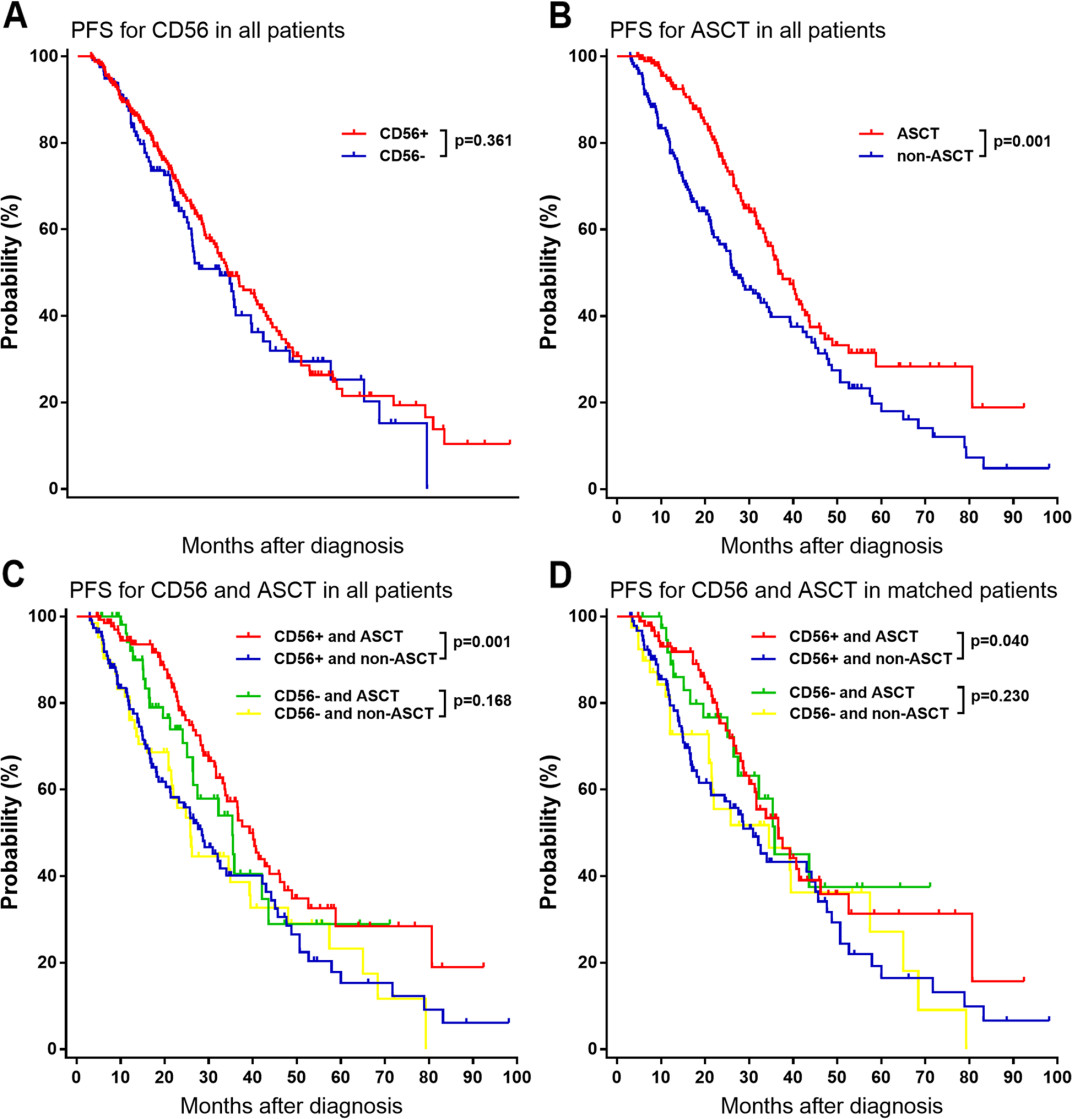
Kaplan-Meier survival curves on PFS of patients with NDMM. **A** all patients. **B** all patients. **C** all patients. **D** matched patients

The median follow-up time for all patients was 30.0 (range 3–114) months. It was showed that ASCT could improve OS and PFS of patients (Fig. 1B and 2B), however, CD56 expression was not related to OS and PFS by Kaplan-Meier survival analysis (Fig. 1A and 2A). Among CD56 positive patients, the median OS were 87.6 (95% CI, 75.6–99.6) months and 56.1 (95% CI, 43.6–68.6) for patients with and without ASCT, respectively (*p* < 0.001, Fig. 1C); the median PFS were 39.3 (95% CI, 34.9–43.7) months and 28.5 (95% CI, 22.1–34.9) for patients with and without ASCT, respectively (*p* < 0.001, Fig. 2C). After matching, CD56 positive patients who received ASCT also had longer OS (87.6 vs.56.1 months, *p* = 0.049) and PFS (36.7 vs.30.9 months, *p* = 0.040) than those CD56 positive patients who had no ASCT (Fig. 1D and 2D). Among CD56 negative patients, the median OS were 49.2 (95% CI, 34.7–63.6) months and 65.0 (95% CI, 42.8–87.2) for patients with and without ASCT respectively (*p* = 0.530, Fig. 1C); the median PFS estimated were 35.4 (95% CI, 27.1–43.7) months and 25.9 (95% CI, 21.8–30.0) for patients with and without ASCT respectively (*p* = 0.168, Fig. 2C). After matching, CD56 negative patients received ASCT also had similar OS (49.2 vs.78.0 months, *p* = 0.564) and PFS (35.8 vs.34.5 months, *p* = 0.230) with those CD56 negative patients who had no ASCT (Fig. 1D and 2D).

## Discussion

In our study, we evaluated the prognostic value of CD56 expression for patients with NDMM undergoing ASCT. We found that ASCT might improve the OS and PFS of CD56 positive patients and had no impact on survival of CD56 negative patients in novel-agent era.

CD56 is a neural cell adhesion molecule associated with the axon growth during normal embryogenesis. It is expressed in most of the malignant plasma cells and is very common on myeloma cells. Several studies showed CD56 expression could be detected in 55 ~ 85% patients with MM [7–13]. Pan Y et al. [7] retrospectively analyzed 50 patients with NDMM and found 74% MM patients with CD56 expression. Skerget M et al. [8] also detected CD56 expression in 110 patients with NDMM and reported that CD56 expression rate was 71%. Another study assessed 34 patients with NDMM and reported that 29 (85.3%) patients had CD56 expression [13]. Our study showed that 67.6% patients presented CD56 expression which was similar with previous studies.

The prognostic value of CD56 expression in NDMM have been assessed in several studies. It was showed that lack of CD56 expression of NDMM was a poor prognostic factor. Pan Y et al. [7] analyzed the prognostic value of CD56 expression in 50 patients with NDMM and found that CD56 was a favorable prognostic factor for OS in multivariate analysis. Moreover, CD56 positive patients had higher overall response rates (ORR) than CD56 negative patients after induction therapy. Skerget M et al. [8] analyzed 110 patients with NDMM and showed that the median PFS of CD56 positive patients was longer than CD56 negative patients. One multicenter study which enrolled 35 patients with NDMM carrying t (14;16) and 124 patients without t (14;16) as a control indicated that lack of CD56 expression was a poor prognostic factor for MM patients with t (14;16) in novel-agent era [17]. However, some studies failed to confirm the result. Mathew P et al. [10] conducted a study of 68 untreated patients with MM from a single institution and found that lack of CD56 expression was not a prognostic factor in MM. One prospective, long-term study enrolled 204 MM patients also found that CD56 expression carried no distinct adverse prognosis [11]. Hundemer M et al. [20] analyzed CD56 expression of patients with NDMM who received ASCT by flow cytometry and indicated that CD56 was not a prognostic factor. Other studies could not also consider CD56 expression as a prognostic factor for MM [19, 21].

At present, ASCT remains the standard treatment after induction therapy for eligible patients with NDMM [2]. Comparing with standard therapy, patients received ASCT had an increase in the CR rate and a longer OS (54 vs. 42 months) [23]. After induction therapy, only half of transplant-eligible patients received ASCT which was mainly due to the fact that transplant-related drugs were not covered by the medical insurance and some patients could not bear the cost. In addition, some patients refused transplantation because they were worried about the adverse effects of ASCT. We also found that ASCT may improve respose rates of CD56 positive and negative patients. It was consistent with previous reported results. There were two studies evaluating prognostic value of CD56 on survival of MM patients undergoing ASCT and they found that CD56 was not related to the outcome of patients received ASCT [20, 21]. In this study, we found that CD56 expression was not related to OS and PFS of NDMM patients by Kaplan-Meier survival analysis. We divided the patients into four groups using CD56 and ASCT, and found that ASCT improved the survival of CD56 positive patients, but did not significantly improve the survival of CD56 negative patients. The result was inconsistent with the conclusions of these two studies. These two studies suggested that CD56 expression was not associated with the survival of patients received ASCT. However, we believed that ASCT could improve the survival of CD56 positive patients but not CD56 negative patients. The reason might be different induction therapy regimens. In our study, all patients received induction therapy combining novel agents followed by single course of melphalan 200 mg/m^2^ as intensive chemotherapy prior to transplant of autologous peripheral blood stem cells. Patients of other two studies received conventional chemotherapy before ASCT. Both of these studies had not enrolled CD56 positive and negative patients who had not received ASCT. They did not evaluate the effect of ASCT on survival of CD56 positive or negative patients with NDMM. In our study, ASCT could significantly prolong OS and PFS of CD56 positive patients. However, ASCT had no impact on survival of CD56 negative patients. It was showed that ASCT may improve the ORR of CD56 positive and negative patients, however, ASCT could not prolong the survival of CD56 negative patients. This may be due to faster progression or poorer response to subsequent treatment in CD56 negative patients. It suggested that NDMM CD56 positive patients may benefit more from ASCT than CD56 negative patients in novel-agent era. CD56 negative patients had advanced clinical features and a high proportion of t (14; 16), which may result in a poor response to ASCT. CD56 is a nerve cell adhesion molecule that mediates the adhesion of myeloma cells to the bone marrow matrix and stromal cells. The decreased adhesion of myeloma cells to the bone marrow matrix and stromal cells in CD56 negative patients may be related to the poor anti-myeloma effect of high dose melphalan.

The limitation of this study was the nature of its retrospective study, which resulted in the inability to effectively control interferon factors. Therefore, prospective randomized controlled studies are needed to further confirm the conclusions of this study.

In conclusion, our study suggested that ASCT could significantly prolong OS and PFS of CD56 positive patients, but had no impact on survival of CD56 negative patients. It needs further study to confirm these results in the future.

## Data Availability

The analyzed data sets generated during the study are available from the corresponding author on reasonable request.
